# Characteristics, Structure, and Effects of an On-Line Tool for Improvement in Adolescents’ *Competency for Interaction With Alcohol*: The e-ALADO^TM^ Utility

**DOI:** 10.3389/fpsyg.2019.00127

**Published:** 2019-02-26

**Authors:** Jesús de la Fuente, Inmaculada Cubero, Francisco Javier Peralta, Mari Carmen Sánchez, Jose Luis Salmerón, Salvatore Fadda

**Affiliations:** ^1^School of Education and Psychology, University of Navarra, Pamplona, Spain; ^2^Department of Psychology, University of Almería, Almería, Spain; ^3^Educational Psychologist, Centro Educativo Agave, Almería, Spain; ^4^Prevention Service, University of Sassari, Sassari, Italy

**Keywords:** prevention of alcohol intake, competence model, adolescence, e-Program, technological utility

## Abstract

This research report aims to present the characteristics, structure and effects of a psychoeducational technological innovation (called the e-ALADO Program) for the prevention of alcohol intake in adolescents. Based on the Competency model for interaction with alcohol, this program consists of a total of 24 lessons that promote conceptual, procedural, and attitudinal learning, in ICT format (www.alado.es). The hypothesis of this validation study established that adolescents treated with the program would improve their levels of competence and their interaction behavior with alcohol, depending of their personal level of self-regulation. A total of 148 adolescents from 12 to 16 years of age from three Spanish educational centers with different sociocultural contexts participated. A quasi-experimental methodology with repeated measures and use of inferential analysis was used (ANOVAs and MANOVAs). The results show a main principal effect of the Treatment variable, of the Self-Regulation Level variable, and an interaction effect of Treatment × Self-regulation in the conceptual and attitudinal subcompetence for interaction with alcohol. The results are discussed in the face of new technological developments that allow the evaluation and intervention in the prevention of alcohol intake with adolescents. An important implication of this work is related to the importance of self-regulation as a psychological variable. Also, the suitability of psychoeducational interventions with new technological formats in the prevention of adolescents’ alcohol intake as entrepreneurial activity.

## Introduction

### Preventing Alcohol Intake Through Online Systems in Adolescence

#### Problems of Alcohol in Adolescence

Alcohol consumption among adolescents is an international problem (Elsayed et al., 2018; Foster et al., 2018; Kendler et al., 2018). In Spain, the age of contact with alcohol and intake of alcohol is increasingly premature. The Spanish Surveys on Drug Use in Secondary Education (Spanish Observatory of Drugs and Drug Addiction [Observatorio Español de la Droga y las Toxicomanías] (OEDT) 2014–2015) indicate that, despite a reduction in consumption rates, alcohol remains the most frequently consumed substance among Spanish adolescents. A high percentage of Spanish adolescents report regularly consuming alcohol and having started consumption at a very early age (Quiroga et al., 2018).

It’s a fact that this problem requires bold and innovative intervention strategies that allow educating pre-adolescents with primary prevention strategies, before the problem requires intervention in secondary prevention. For this purpose, recent research has tried to show whether treatments based on ICT systems are more efficient than traditional systems (Bennett and Glasgow, 2009; Munson and Jaccard, 2018). This question is especially relevant for adolescent populations, which can already be considered as a digital natives (Collins and Halverson, 2009).

#### The Importance of the Use of Online Technological Strategies

The use of online ICTs has been increasing in recent years, based to their versatility and their numerous possibilities for addressing different populations (White et al., 2010; Sundström et al., 2017; for more information, see the present Monographic). There have been quite a number of web-based interventions in adult populations (Johansson et al., 2017; Mellentin et al., 2017; Baldin et al., 2018; Han et al., 2018). Online interventions have also been carried out in populations of university students and youth (Bertholet et al., 2015; Berman et al., 2016; Fazzino et al., 2017).

However, utilities oriented to adolescent populations are much fewer in number (Arnaud et al., 2016; Caudwell et al., 2016). Moreover, many such online tools are based on psychosocial or clinical models of prevention, applied to the sphere of educational psychology—despite the recent development of theoretical and research models using the *Competency Approach of Educational Psychology* itself, and the evidence that has upheld the value of this approach (de la Fuente et al., 2017a). This Competency Approach assumes the importance of the acquisition of three levels of learning: knowledge, skills, and attitudes, in a manner consistent with the Gagné Educational Psychologist model (Gagné, 2013). Consequently, there is a need for evidence-based, online technological developments with an educational psychology approach. This aim of this paper is to present the characteristics and effects of the ALADO online utility, a technological tool that has already been tested in educational psychology interventions for the prevention of alcohol intake in adolescents. Other complementary results have already been published, in Spanish (Marcos, 2013).

### The e-ALADO Utility as a Psychoeducational Technological Innovation

#### Foundations

##### The theory of Self- vs. Externally-Regulated Learning^TM^ and the alcohol intake

Brown (1998) conceptualized *self-regulation* (SR) from the tendency of individuals regarding their particular ability to plan and flexibly manage their behavior. SR has received particular attention in recent years as an essential factor to better comprehend health and disease, as a result of healthy habits and individuals’ capacity to set and maintain healthy goals (Mann et al., 2013; Kitsantas et al., 2017). In fact, SR has been considered, based on recent evidence, as a variable or construct of the meta-behavioral order, meaning, a meta-skill or skill to manage the cognitive, affective, and motivational abilities.

This *Self-Regulated Learning vs. Externally-Regulated Learning* (SRL vs. ERL) theoretical approach (de la Fuente, 2017) predicts that the individuals’ may be characterized on a behavioral continuum: *Self-regulatory, A-regulatory, or Dys-regulatory behavior.* In this case, despite the fact that the name of the Theory refers to Self-Regulated Learning (SRL), the construct it refers to is that of personal self-regulation (SR) as a more general construction, considering that SRL is a specific type of Self-Regulation. There is evidence that people may have different degrees of personal self-regulation (high–medium–low), alluding to the extent and to the number of practices they employ to exercise their health behavioral regulation (Zimmerman et al., 1999; Clark et al., 2001). *Self-regulation* (SR), *or high in self-regulation*, may be considered as the degree of a person’s *positive* *proactivity* in its active and adequate management of the regulation of well-being and health (Brown, 1998). *A-regulation* (AR), *or medium in self-regulation*, may be defined conceptually as the lack of proactivity and so equivalent to the concept of behavior *reactivity* (Zimmerman and Labuhn, 2012). *Dys-regulation* (DR), *or low in self-regulation*, may be defined as the degree of *negative proactivity*, that is, of active and inadequate management to regulate one’s behavior. This de-regulation avoids the effort involved in proactive self-regulation of health, and of the procrastination (Clariana, 2013; Balkis and Duru, 2017).

This theoretical model considers the context as the set of situational stimuli that can make probable the directionality of a behavior, in interaction with the subject, being these of real, virtual or symbolic type. (1) *External Self-Regulatory context (ESR)* promotes positive or adequate proactivity, or clearly fosters self-regulation. In this context, there are numerous external signs or encouragements which promote and make self-regulated behavior more likely at the beginning, during, and at the end of all behavioral acts. Highly predictable of positive events are a feature of this context. (2) *External A-Regulatory* (EAR) *context* does not promote external self-regulation, or de-regulation. In this context, there are no external signs or encouragements to make self-regulated behavior or de-regulated behavior more likely at the beginning, during, or at the end of the action. Highly unpredictable events are a feature of this context. (3) *External Dys-regulatory* (EDR) context, actively promoting de-regulation. The context promotes non-positive, inadequate, or negative proactivity. In this context, there are many external signs which make de-regulated behavior more likely, favoring active de-regulation at the beginning, during, and at the end of the behavioral act. This kind of context means that the individual needs to make a great effort to engage in self-regulation. Highly predictable of negative events are a feature of this context. In a previous research report, examples of this theoretical formulation were presented (de la Fuente, 2017; pp. 3–4).

#### Characteristics

The e-*Alado* is a technological online utility^1^, based on the ALADO program (de la Fuente et al., 2012), designed for adolescents The program provides assessment and intervention in different matters of learning. It can be used by students, teachers, and parents, but in this case it was applied only to students. The teacher’s guide and instructions for use have been published.

#### Structure

The *competency model for interacting with alcohol* (de la Fuente et al., 2017a) is the foundation of this utility and its associated program, and is consistent with the *SRL vs. ERL* Theory (de la Fuente, 2017). Its three constituent types of learning (subcompetencies) have been defined theoretically. Prior research has shown the importance of each type of learning (de la Fuente et al., 2017a):

(1) *Knowing* (understanding or conceptual subcompetence), that is, having adjusted knowledge and not only information with reference to a given phenomenon, with facts, concepts and principles that have been well elaborated:

1. Knowing the properties, characteristics and effects of alcohol.
2. Being able to scientifically explain the effects of alcohol at the neuronal, personal and social levels.
3. Knowing principles and rules that regulate the use of alcohol in our context.

(2) *Being able to* (know-how or procedural subcompetence), referring to the meta-skill of self-regulation and decision-making, and to social interaction skills for behaving as one wishes:

1. Knowing how to make the right choices in our culture’s typical interaction with this substance.
2. Knowing how to exercise counter-control in the face of social pressure.
3. Knowing how to self-regulate one’s behavior in general, and particularly one’s alcohol intake.
4. Knowing how to use adjusted coping strategies, when facing personal problems.

(3) *Mindset* (knowing how to be or attitudinal subcompetence), referring to attitudes, values and habits toward alcohol as a substance:

1. Having a critical attitude toward certain inadequate social behaviors and habits.
2. Incorporating the value of respect for oneself and care of one’s body in recreational situations.
3. Having healthy alternative habits that help in preventing alcohol intake.

In this Competence model for interaction with alcohol, it is assumed that: (1) a person is *competent*, when they have knowledge (know), have skills and meta-skills (know how to do) and want (know how to be); (2) the level of competence predicts the way in which the adolescent *interacts* with alcohol, that is, how he manages and decides in situations where alcohol is present and is capable of exercising counter-control and behavioral self-regulation, and finally, do not consume alcohol. It is assumed that there is a good interaction with the substance when it is not consumed during adolescence.

##### Teaching–learning content

The teaching–learning content of the e-ALADO utility is as follows (see the demo^2^):

(1) *Facts, concepts, and principles content* (see Anex, Figure A1).

• Facts from real life (cognitive branch of knowledge):
  Alcohol in human culture
  Situations in which alcohol is present
  Personal and social consequences of inadequate alcohol use
• Concepts referring to alcohol (cognitive branch of knowledge):
  Brain effects from alcohol intake 1
  Brain effects from alcohol intake 2
  Brain effects from alcohol intake 3
  Brain effects from alcohol intake 4
• Principles of behavior (affective branch of knowledge; beliefs):
  Principle of healthy recreation
  Principle of health and alcohol abstinence
  Principle of self-regulation and responsibility (self-determination)

(2) *Procedures content:* Meta-skills and skills (see Anex, Figure A2).

• Personal:
  Improving as a person
  Self-regulation and personal self-determination (1)
  Self-regulation and personal self-determination (2)
  Self-regulation and personal self-determination (3)
  Strategies for coping with problems (1)
  Strategies for coping with problems (2)
• Social-moral
  Assertiveness and social skills
  Values clarification
• Cognitive-linguistic
  Making decisions about how to behave in complex situations
  Imagining alternatives in complex situations

(3) *Attitudes, values, and rule-following (habits) content* (see Anex, Figure A3).

• Attitudes:
  Exercising caution with alcohol
  Recreation under control
  Responsibility
• Values:
  Personal honesty
  Respect and care for one’s body
  Health and well-being
• Habits:
  Habits of practicing sports
  Habits of recreational activities
  Habits at parties and celebrations
  Habits at difficult personal times

#### Methodology and Activities

The program was carried only with on-line activities of the same. The teacher-tutor only motivated the students to do so and after the activity dialog about the doubts. The duration was 8 months because the weekly time of the tutorial task to do it was only 1 h. Also to give time to a consistent attitudinal change and not remain as a punctual and intensive activity, without impact on adolescents. Eight learning modules over 8 months (October–May). Each month-long learning module includes three learning units of subcompetencies, corresponding to the conceptual, procedural, and attitudinal levels. These eight modules incorporate initial, process and product assessments, corresponding to the teaching–learning process. For each of the 24 lessons (three lessons per month), 8 for each conceptual, procedural, and attitudinal subcompetency (see content structure), there are different types of activities:

(1) *Initial assessment*. Reflection on what you know about that particular learning point.
(2) *Motivation activities*. Finding value and meaning in the learning point.
(3) *Learning activities*. Learning new concepts, procedures or personal habits.
(4) *Consolidation and generalization activities*. More activities to learn more and behave better in more situations.
(5) *Extension activities*. Finding answers to your unresolved questions.
(6) *Support activities*. Reinforcement of what has been learned.
(7) *Final assessment*. Self-assessment of what has been learned in the lesson.
(8) Scoring.

An example of the activities developed, in each lesson, could be found in the Teacher’s Guide of Intervention (only in Spanish)^3^.

### Objectives and Hypotheses

Based on the foregoing assumptions, the *objectives* of this report were: (1) to explain the structure of the e-ALADO technological innovation; (2) gather evidence about its effects, following implementation. Our *hypotheses* established that the application of this online program: (1) would bring about a statistically significant increase in students’ levels of conceptual, procedural, and attitudinal subcompetencies of interaction with alcohol, and improvement in their actual interaction with alcohol, compared to their prior levels (before program application); (2) This improvement would be moderated by students’ pre-existing level of personal self-regulation, according to the exposed model and the existing theoretical evidence (de la Fuente et al., 2017a).

## Materials and Methods

### Participants

The population under study were students from public secondary schools in a southern province of Andalusia (Spain). To gain variability in the sample, participants were not selected or grouped by any previous criteria (consumption, problems with alcohol). The sample was the natural one existing in the three centers. In order to include different types of schools in this investigation, schools were selected from three types of urban areas: (1) center of town, from a medium-high social stratum (*n* = 106), (2) surrounding neighborhoods, from a medium-low social stratum (*n* = 101) and (3) outlying, marginalized population areas with compensatory education, from a low social stratum (*n* = 121). The homeroom teachers from every group participated voluntarily in the experiment, having been invited by the School Psychology adviser of the local Teacher Development Center. Initial participating students were between the ages of 12 and 17 years [12 (*n* = 51), 13 (*n* = 80), 14 (*n* = 97), 15 (*n* = 55), 16 (*n* = 35), and 17 (*n* = 10)] and were enrolled in compulsory secondary education (grades 7–10) at one of three public secondary schools. This age range was selected for methodological purposes, making it possible to form two groups, the 12- to 14-year-olds (*n* = 207), corresponding to the first stage of adolescence (puberty, or early adolescence), and the 15- to 17-year-olds (*n* = 121), corresponding to the second stage of adolescence (adolescence *per se*). The final sample size from which all measurements (pre–post) were taken contained 148 subjects. Of these, 80 were male (54% of the sample) and 64 were female (46% of the sample). The mean age of the sample was 13.82 ± 1.19 years old.

### Instruments

#### Conceptual Subcompetency

The scale *Evaluación de los Hechos, Conceptos y Principios sobre el Alcohol, EHCP* (Assessment of facts, concepts, and principles about alcohol, AFCP) was used (Cubero and Sánchez, unpublished). The scale is composed of 38 items concerning the effects of alcohol use; psychometric analyses of this scale show reliability (α = 0.827) and consistent construct validity, with three factors: knowledge of facts, concepts, and principles concerning alcohol. Exploratory Factor Analysis (EFA) showed KMO = 0.801; Bartlett’s Sphericity Test (df = 703) = 2767.595; *p* < 0.001. Confirmatory Factor Analysis (CFA) showed adequate indicators for the Default model: *Chi-square* = 1612.957, *Degrees of freedom* (779–117): 662, *p* < 0.001; all the variances are significant for *p* < 0.001; NFI = 0.865; RFI = 0.848; IFI = 0.914; TLI = 0.902; CFI = 0.913; RMSEA = 0.025; HOELTER model = 1095 (*p* < 0.05), 1138 (*p* < 0.01).

#### Procedural Subcompetency

The *SRQ*, *Self-Regulation Questionnaire* (Brown et al., 1999; Carey et al., 2004; Neal and Carey, 2005) was used, in its 21-item abbreviated Spanish version, SRQ-21 (Pichardo et al., 2018). Its reliability (α = 0.826) and validity values are consistent, with two dimensions, planning and action control. EFA showed an index KMO = 0.985; Bartlett’s Sphericity Test (df = 210) = 3603.882; *p* < 0.001. CFA showed adequate indicators for the Default model: *Chi-square* = 408.448, *Degrees of freedom* (252–64):188, *p* < 0.001; all the variances are significant for *p* < 0.001; NFI = 0.894; RFI = 0.870; IFI = 0.940; TLI = 0.925; CFI = 0.9393; RMSEA = 0.022. However, this structure does not concur with others found in other samples (Pichardo et al., 2014; Artuch-Garde et al., 2017).

#### Attitudinal Subcompetency

The scale for *Evaluación de las Actitudes* *ante el alcohol, EAA* [Assessment of Attitudes toward Alcohol, AAA] was used (Cubero and Sánchez, 2018). A total of eight items assess attitudes and values toward alcohol (α = 0.825). EFA showed KMO = 0.869; Bartlett’s Sphericity Test (df = 28) = 529.335, *p* < 0.001. CFA showed adequate indicators for the Default model: *Chi-square* = 59.274, *Degrees of freedom* (44–24): 20, *p* < 0.001; all the variances are significant for *p* < 0.001; NFI = 0.913; RFI = 0.926; IFI = 0.934; TLI = 0.918; CFI = 0.924; RMSEA = 0.08; HOELTER model = 1215.

**FIGURE 1:**
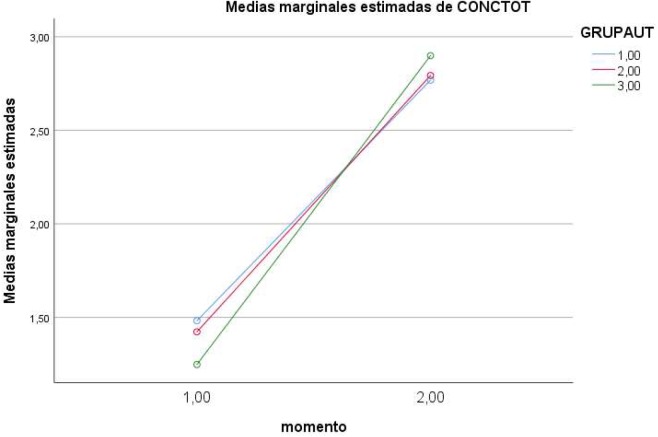
Effects of Moment × Level of regulation on knowledge (1 = low; 2 = medium; 3 = high).

**FIGURE 2:**
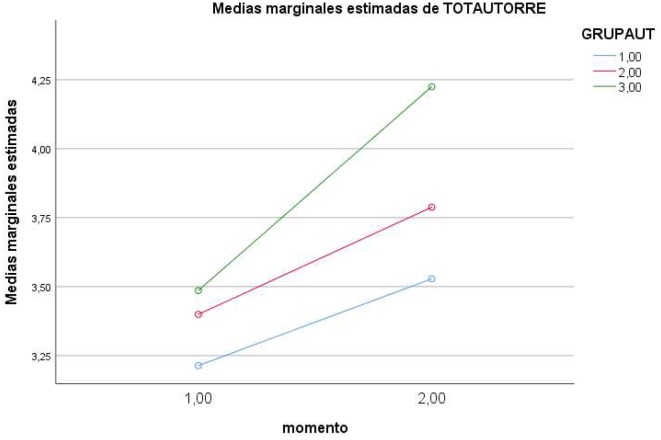
Effects of Moment × Level of regulation on self-regulation (1 = low; 2 = medium; 3 = high).

**FIGURE 3:**
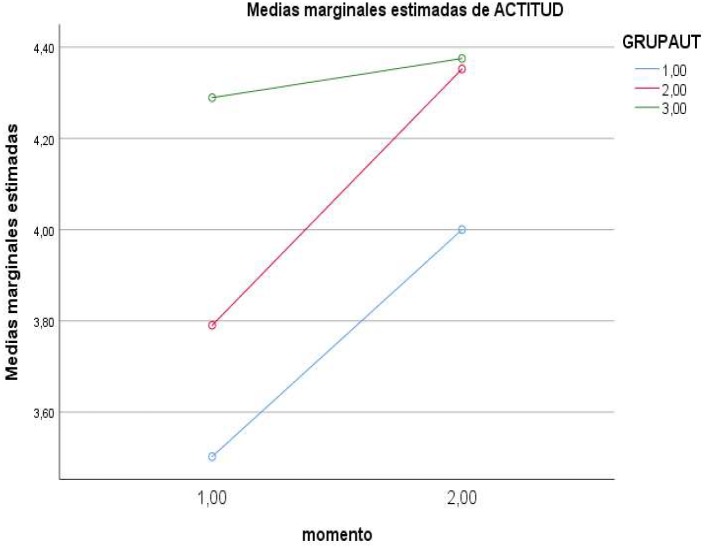
Effects of Moment × Level of regulation on attitudes (1 = low; 2 = medium; 3 = high).

**FIGURE 4:**
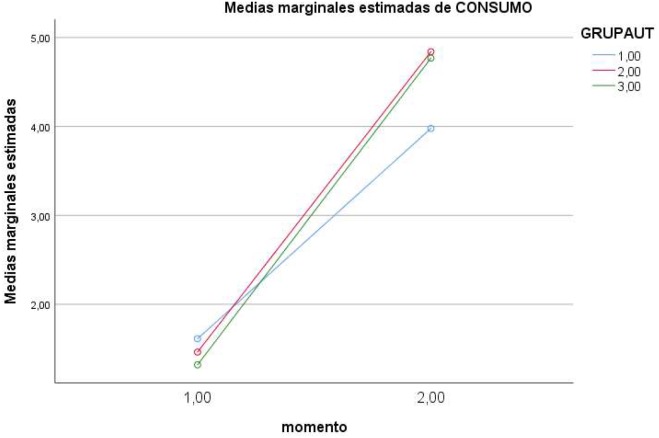
Effects of Moment × Level of regulation on an adjusted interaction with alcohol (1 = low; 2 = medium; 3 = high).

In the case of the *interaction of Moment* ×*Level of self-regulation*, partial effects showed a significant effect on the dependent variable *knowledge* [*F*(2,184) = 4.661, *p* < 0.01 (Pillai’s trace), η = 0.050, power = 0.775], and on *interaction with alcohol* [*F*(2,184) = 6.543, *p* < 0.01 (Pillai’s trace), η = 0.068, power = 0.908]. Direct values are presented in Table 1 and the effects in Figure 1–4.

**Table 1:** Conceptual continuum and typologies of each Self-Regulatory Behavior (reproduced with permission).

Characteristics of the person	Self-Regulation (SR) High–moderate–low POSITIVE PRO-ACTIVITY (+1)	A-Regulation (AR) No regulation RE-ACTIVITY (0)	Dys-Regulation (DR) Low–moderate–high NEGATIVE PRO-ACTIVITY (-1)
	*Before* Self-analysis of tasks Self-defines goals Self-motivation	*Before* No analysis of tasks No goals No motivation	*Before* Erroneous self-analysis Erroneous goals Self-demotivation
	*During* Self-observation Self-analysis Self-correction	*During* No self-observation No supervision No self-correction	*During* Self-distraction Cognitive self-avoidance Self-impediment procrastination
	*After* Self-reflection Self-attributions Positive self-affects	*After* No reflection No attributions No affects	*After* Erroneous self-assessment Erroneous self-attributions Negative self-affect
**Type of activity**	**Self-Regulatory (SR) High–moderate–low PRO-ACTIVITY (+)**	**A-Regulatory (AR) No regulation RE-ACTIVITY (=)**	**Dys-Regulatory (DR) Low–moderate–high PRO-ACTIVITY (-)**
Academic	Self-regulated learning	No norms/limits	Self-induction impediment
Road safety	Self-regulation in driving	No norms/limits	Self-induction of risks
Health	**SR in health^∗^**	**No norms/limits^∗^**	**Self-induction of excesses^∗^**
TV	SR in TV	No norms/limits	Self-induction of excesses
Family	SR in family	No norms/limits	Self-induction of risks
Technology of Information and Communication (TIC)	SR in TIC	No norms/limits	Self-induction of excesses
Sexual	SR in risky sexual behavior	No regulation	Self-induction of risks
Violence	SR in harmonious relations	No norms/limits	Self-induction of excesses
Spouse/partner	SR in interaction	No regulation	Self-induction of excesses


*^∗^Place of alcohol intake in this theoretical model.*

#### Adjusted Behavior in Interacting With Alcohol

We used the *Escala de Ajuste en la interacción con el alcohol* [Scale of Adjustment in interacting with alcohol], which contains four items (α = 0.915). This scale belongs to the *Inventario de Evaluación de conocimientos, actitudes e interacción con el alcohol* (Cubero and Sánchez, unpublished) [Inventory for Assessment of knowledge, attitudes and interaction with alcohol].

All instruments were validated with Spanish teenagers. The data and validation procedure of these instruments have already been presented in a previous research report (de la Fuente et al., 2017a).

### Procedure

Data collection instruments were applied over the course of the school year 2009–2010, within the framework of the *Alado Project of Excellence* (2007–2010), through an online utility created for this purpose (see footnote 1). Although these data are old, we consider that, given the characteristics of them, they still have a great importance for the variables analyzed (see Table 2).

**Table 2:** Sequence of the e-ALADO intervention.

• INITIAL ASSESSMENT (OCTOBER).
**October** • UD01. Program Presentation (C) • UD02. Self-regulation and personal self-determination, before, 1 (P) • UD03. Exercising caution with alcohol (A)
**November** • UD04. Drinks that contain alcohol (C) • UD05. Self-regulation and personal self-determination, during, 2 (P) • UD06. Recreation under control (A)
**December** • UD07. Health consequences of alcohol (C) • UD08. Self-regulation and personal self-determination, after, 3 (P) • UD09. Responsibility (A)
**January** • UD10. Brain effects from alcohol intake 1 (C) • UD11. Coping strategies 1 (P) • UD12. Personal strength (A)
**February** • UD13. Brain effects from alcohol intake 2 (C) • UD14. Coping strategies 2 (P) • UD15. Respect and care for one’s body (A)
**March** • UD16. Brain effects from alcohol intake 3 (C) • UD17. Assertiveness and social skills 1 (P) • UD18. Health and well-being (A)
**April** • UD19. Brain effects from alcohol intake 4 (C) • UD20. Assertiveness and social skills 2 (P) • UD21. Habits of practicing sports and recreational activities (A)
**May** • UD22. Principle of health and alcohol abstinence (C) • UD23. Values clarification and decision-making in complex situations (P) • UD24. Habits at celebrations and in difficult personal times (A)
• FINAL ASSESSMENT


*C, conceptual subcompetence; P, procedural subcompetence; A, attitudinal subcompetence.*

Cooperation had been previously requested from the Teacher Development Center, from the students’ parents and from the School Board, for student participation. The project was approved by the *University Bioethics Commission* (University of Almería; Ref. 2009; n° 028) and by the *School Boards* of the participating schools. The students participated voluntarily. The parents were informed in writing. As the participants in the Project were minors, both the parents and school administrators gave written informed consent for the study. The consent obtained was both informed and written. The data was protected in an archived and registered file, as indicated by the Spanish Data Protection Law.

### Data Analyses

An *quasi-experimental single-group study*, with repeated measures pre and post, was used. The independent variables considered were (1) Moment (Treatment) × (2) Level of Self-regulation. For levels of the independent variable self-regulation, cluster analysis was used, obtaining three levels: low, medium, and high. Inferential statistical analyzes (multivariate analysis, ANOVA) were carried out using SPSS (v.23.0 for Windows) to verify the effect of the independent variables on the dependent ones: the level of competence.

## Results

### E-ALADO Program Effects

There was a significant main effect of the *Moment* factor [*F*(4,175) = 270.866, *p* < 0.001 (Pillai’s trace), η = 0.861, power = 1.0; moment 2 > 1, *p* < 0.001], of *Level of regulation* [*F*(8,352) = 3.497, *p* < 0.001 (Pillai’s trace), η = 0.074, power = 0.981; moment 2 > 1, *p* < 0.001], and of the *Interaction* of these two [*F*(8,352) = 3.497, *p* < 0.001 (Pillai’s trace), η = 0.074, power = 0.981] on the set of dependent variables being analyzed (see Table 3).

**Table 3:** Effect of the e-ALADO utility in interaction with students’ level of self-regulation.

	Moment	Self-regulation level				*Post hoc*
		Low (*n* = 51)	Medium (*n* = 62)	High (*n* = 35)	Total (*n* = 148)	
Knowledge	1	1.48 (0.34)	1.42 (0.27)	1.24 (0.20)	1.40 (0.29)	
	2	2.76 (0.34)	2.79 (0.12)	2.89 (0.58)	2.82 (0.20)	2 > 1^∗∗^
	Total	1.71 (0.60)	1.62 (0.55)	1.71 (0.77)	1.68 (0.63)	n.s.
Procedures	1	3.21 (0.69)	3.39 (0.38)	3.48 (0.24)	3.35 (0.50)	
	2	3.52 (0.51)	3.78 (0.32)	4.22 (0.47)	3.87 (0.52)	2 > 1^∗∗^
	Total	3.26 (0.67)	3.45 (0.40)	3.69 (0.46)	3.45 (0.54)	H > L^∗∗^ H > M^∗^
Attitudes	1	3.50 (1.01)	3.79 (0.83)	4.28 (0.84)	3.80 (0.94)	
	2	4.00 (1.00)	4.35 (0.74)	4.37 (0.65)	4.25 (0.88)	2 > 1^∗∗^
	Total	3.59 (1.00)	3.83 (0.83)	4.31 (0.78)	3.89 (0.94)	H > L^∗∗^ H > M^∗^
Interaction	1	1.58 (0.75)	1.46 (0.68)	1.33 (0.52)	1.47 (0.67)	
	2	3.39 (1.03)	4.84 (0.37)	4.76 (0.38)	4.54 (0.85)	2 > 1^∗∗^
	Total	1.97 (0.89)	1.93 (0.53)	2.16 (0.45)	2.01 (0.76)	n.s.


*^∗^*p* < 0.05, ^∗∗^*p* < 0.001.*

Partial analysis showed the effects more precisely. The *Moment* factor showed a significant partial effect on each of the variables analyzed, with improvement registered in levels of variables; in *knowledge* [*F*(1,184) = 773.523, *p* < 0.001 (Pillai’s trace), η = 0.813, power = 1.00; moment 2 > 1, *p* < 0.001]; in *self-regulation* [*F*(1,184) = 27.602, *p* < 0.001 (Pillai’s trace), η = 0.134, power = 0.993; moment 2 > 1, *p* < 0.001]; in *attitude* [*F*(1,1784) = 5.515, *p* < 0.025 (Pillai’s trace), η = 0.028, power = 0.993; moment 2 > 1, *p* < 0.001]; and in *interaction with alcohol behavior* [*F*(1,184) = 5.515, *p* < 0.025 (Pillai’s trace), η = 0.028, power = 0.993].

The *level of self-regulation* factor showed a differential, significant partial effect on each of the variables analyzed. Regarding *knowledge*, no significant effect appeared [*F*(2,184) = 0.349, *p* < 0.706, ns (Pillai’s trace), η = 0.004, power = 0.109]; on *self-regulation* [*F*(2,184) = 9.444, *p* < 0.001 (Pillai’s trace), η = 0.096, power = 0.976; *post hoc*: H > L, *p* < 0.001; H > M, *p* < 0.05]; on *attitude* [*F*(2,184) = 3.974, *p* < 0.02 (Pillai’s trace), η = 0.043, power = 0.765; *post hoc*: H > L, *p* < 0.001; H > M, *p* < 0.05]; on *interaction with alcohol behavior* [*F*(2,184) = 2.749, *p* < 0.05 (Pillai’s trace), η = 0.030, power = 0.537; *post hoc*: n.s.].

### Summary

The experimentally manipulated variable, *e-ALADO Program*, has shown a significant effect in the increase of conceptual, procedural, and attitudinal learning, as well as abstinence (no alcohol consumption). The variable manipulated by selection, the *Self-Regulation Level* of the students, has produced a differential effect on these variables, especially in attitudinal change. There is evidence that, although high students in SR benefit more from the program, this educational experience benefits everyone.

## Discussion

### Foundation and Structure

Following the conceptual scheme of the I + D + I value chain (de la Fuente et al., 2018a), the e-ALADO program can be considered as a Psychoeducational Technological Development with two benefits. The first, which is a conceptual consequence of assuming:

(1) *Psychoeducational Model of Competence*, applied to education. The competence model for the adequate interaction with alcohol (de la Fuente et al., 2017a) assumes that the person must have incorporated the knowledge (facts, concepts, and principles), the procedures (skills and meta-skills of self-regulation) and attitudes (attitudes, values, and appropriate habits). This conception understands that it is necessary to evaluate to know the level of the student in each of the three subcompetences, before and after the intervention: conceptual, procedural, and attitudinal learning. Also, it assumes that an intervention can produce differential effects in each type of subcompetition and it is necessary to know it.
(2) *SRL vs. ERL Theory.* The importance of self-regulation as an individual psychological variable, modulating the effect of the program itself. Recent research is showing that the self-regulation (SR) of the subjects is a meta-behavioral construct that regulated the learning of other behaviors. For example, its role in the behavior of students’ motivational-affective variables has been evidenced (de la Fuente et al., 2017b). Also, its mediating role on the effects of Mindfulness Program has been reported (de la Fuente et al., 2018b).

The second, which is a program in on-line format, which involves more interactivity and empowerment of meaningful learning in students, when addressing a problem of great importance in adolescence: the early prevention of alcohol consumption.

From the point of view of psychological practice, it is important to understand that educational evaluation and intervention programs – whenever possible – must evolve toward on-line technological supports and formats. Technological innovation is a necessity in the field of psychological evaluation and intervention (de la Fuente et al., 2018c; Garaigordobil and Martínez-Valderrey, 2018).

### Effects of Implementation

The *hypotheses* established that the application of this online program: (1) would bring about a statistically significant increase in students’ levels of conceptual, procedural, and attitudinal subcompetencies, and improvement in their level of interaction with alcohol, compared to their prior levels (before program application); (2) This improvement would be moderated by students’ pre-existing level of personal self-regulation, according to the exposed model and the existing theoretical evidence (de la Fuente et al., 2017a).

The results consistently confirmed -although with restrictions- certain of our hypothesis for the prevention of alcohol consumption in adolescence (Spear, 2015; Stigsdotter-Ekberg et al., 2016). Regarding *Hypothesis 1*, that the intervention would bring about a statistically significant increase in students’ levels of conceptual, procedural, and attitudinal subcompetencies, and improvement in their actual interaction with alcohol, compared to their prior levels, the main effect results show an effect of Moment (that is, the effect of having used the utility). These results are partially maintained for the conceptual, procedural, and attitudinal subcompetencies, as well as for interaction with alcohol. The results contribute empirical evidence that the e-ALADO *technological* *utility* helps improve adolescents’ competency for interaction with alcohol, as well as their actual interaction. This result is consistent with data that was presented for the model of alcohol intake competence, referred to the levels of conceptual, procedural, and attitudinal subcompetence are necessary and predict the level of interaction with alcohol (de la Fuente et al., 2017a).

Regarding *Hypothesis 2*, the results also produced evidence that students’ low–medium–high *level of self-regulation* has its own weight in the general effect, just as is established in *SRL vs. ERL Theory* (de la Fuente, 2017). The level of self-regulation determined, by itself, significant differences in the level of procedural and attitudinal subcompetences, but in interaction with the treatment had a significant effect on conceptual subcompetences and the degree of adequacy of interaction with alcohol (or zero consumption). In other words, students’ level of self-regulation (high vs. medium vs. low) would interact with the effect of the utility on the students. Just as the theory predicts, students who are *high in self-regulation* (self-regulating) would have greater likelihood of benefitting from the utility, while students *low in self-regulation* (dysregulatory) would be the least likely to benefit. This fact is consistent with prior research showing the importance of self-regulation as a meta-behavior that is involved in alcohol intake behavior (de la Fuente et al., 2017a) and the health (Garzón-Umerenkova et al., 2018). After the intervention, the participating students showed a significant increase in the conceptual competency (adjustment in the level of facts, concepts and principles related to alcohol intake), but the largest increase occurred in students with a higher level of self-regulation. That also happened with attitudinal subcompetence. Likewise, a significant increase was produced in the procedural competency (personal self-regulation). This conceptual improvement is interactive with students’ level of personal self-regulation. In other words, students who already have a higher level of personal self-regulation get more benefit from this learning experience. There is a significant decrease in the level of contact with alcohol (absence of intake), in comparison to the initial level.

Despite the findings of this study, we must analyze its *limitations*, so that these may be addressed in future research. The first limitation refers to the design: for reasons outside the researchers’ control, it was not possible to include several equivalent control groups. This fact recommends caution in generalizing the results and is an inherent threat to the study’s validity. For this reason, future research studies should revalidate the impact of this technological utility.

## Conclusion

Based on the foregoing, it seems reasonable to foster this type of technological utility in preventive, educational intervention where programs are established to develop personal self-regulation (Castillo and Dias, 2009; Castillo et al., 2012). Training should address the two sectors of the educational community that are directly involved, that is, both teachers and students, in compulsory and post-compulsory secondary education (Sánchez et al., 2007), as well as at university (Mauri et al., 2009). The e-ALADO utility is ready to be transferred to groups who work with adolescents in the prevention of alcohol intake, because it is well founded and has been validated with encouraging results. Given that the utility was produced at a public university, within a publically funded Research Project, the means of transfer is through R&D contracts with public or private organizations and institutions. Another desirable future line of development would be to adapt the utility to other technological formats that are more familiar to adolescents, such as Android or tablets.

However, we can not assume that the e-ALADO program has a linear learning effect, the same for all adolescents. Although the trend found is an effect of general improvement, it is very important to know and pay attention to adolescents with a low level of self-regulation. According to the results, we know that they are students that need more help and external regulation. Also, probably, those who benefit least from this program.

### Practical Implications

Consequently, from the above information, several practical implications are deduced to apply this program with adolescents and Spanish centers:

(1) It’s important to have a minimum previous *teacher training* in the Competency model and in the Program Use Guide, which has not been done in our experimentation. This would improve the involvement of teachers in the program. It is not enough to provide a technological tool to apply it in a decontextualized way.
(2) It is necessary to involve more *families.* Parents should use this e-ALADO Program. Thus, the impact of the community would exert an effect of external regulatory context, ceasing to be an a-regulatory or dys-regulatory context.
(3) It is very important that the Educational Administrations and Centers have an attitude of *engagement* to this problem and the possibilities of change through this program. Our results show that it is worth applying this type of programs in a universal preventive strategy.
(4) Based on the evidence provided by previous research, the best time to apply this Program, as a primary prevention strategy, should be in the last stage of *Primary Education*, in high-risk centers (10–12 years) or in the first stage of *Secondary Education*, in other centers (12–14 years). The Educational Psychologist, as a professional, must assess it in each case. If it’s applied very early, it may be counter-preventive due to the excess of information provided. But if it is applied too late, the impact will be minimal, especially for the change of habits and attitude.

## Author Contributions

JdlF design, analysis, and writing. IC ALADO Project director and text revision. FP center implementation and material design. MS data analysis and text review. JS implementation in centers. SF review of the preventive foundation of intervention.

## Conflict of Interest Statement

The authors declare that the research was conducted in the absence of any commercial or financial relationships that could be construed as a potential conflict of interest.

## Footnotes

1 http://www.alado.es

2 http://alado.es/leccion_demo.html

3 https://www.researchgate.net/publication/319998580_ALADO_GUIA_DE_INTERVENCION
