# Supplementary material for: Characteristics, Structure, and Effects of an On-Line Tool for Improvement in Adolescents’ Competency for Interaction With Alcohol: The e-ALADOTM Utility
Source: Front Psychol. 2019 Feb 26;10:127. doi: 10.3389/fpsyg.2019.00127 (PMC6399204; doi:10.3389/fpsyg.2019.00127)
Supplement: Supplementary file 1 [file Data_Sheet_1.pdf]

ANEX (SPANISH VERSION ONLY)

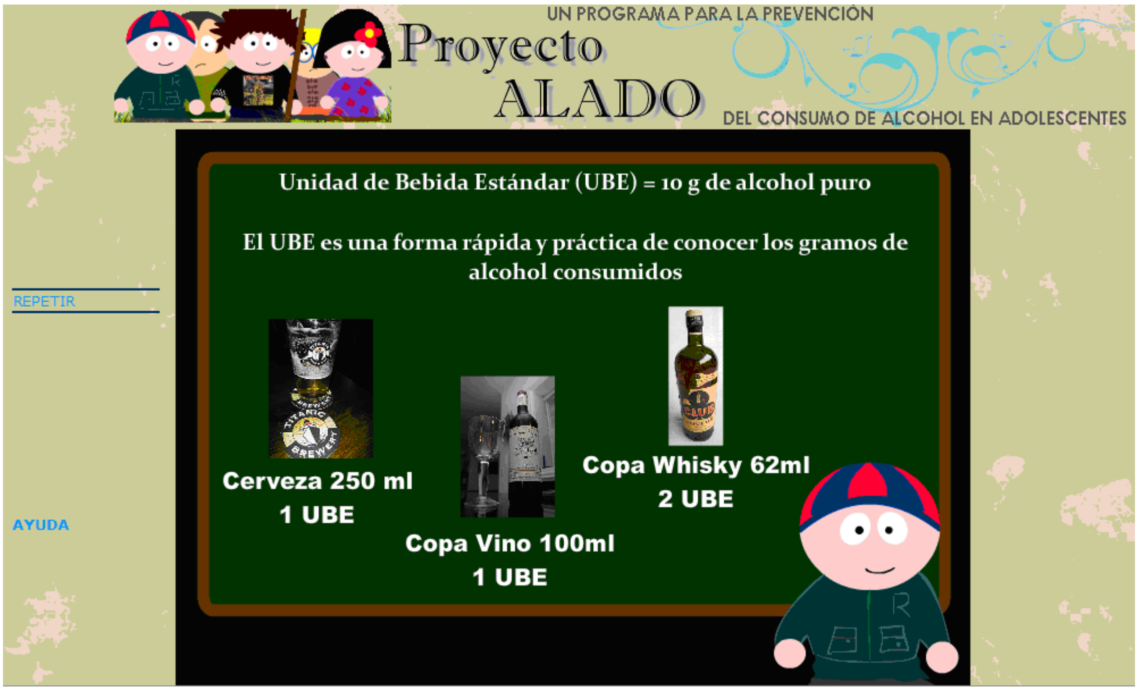

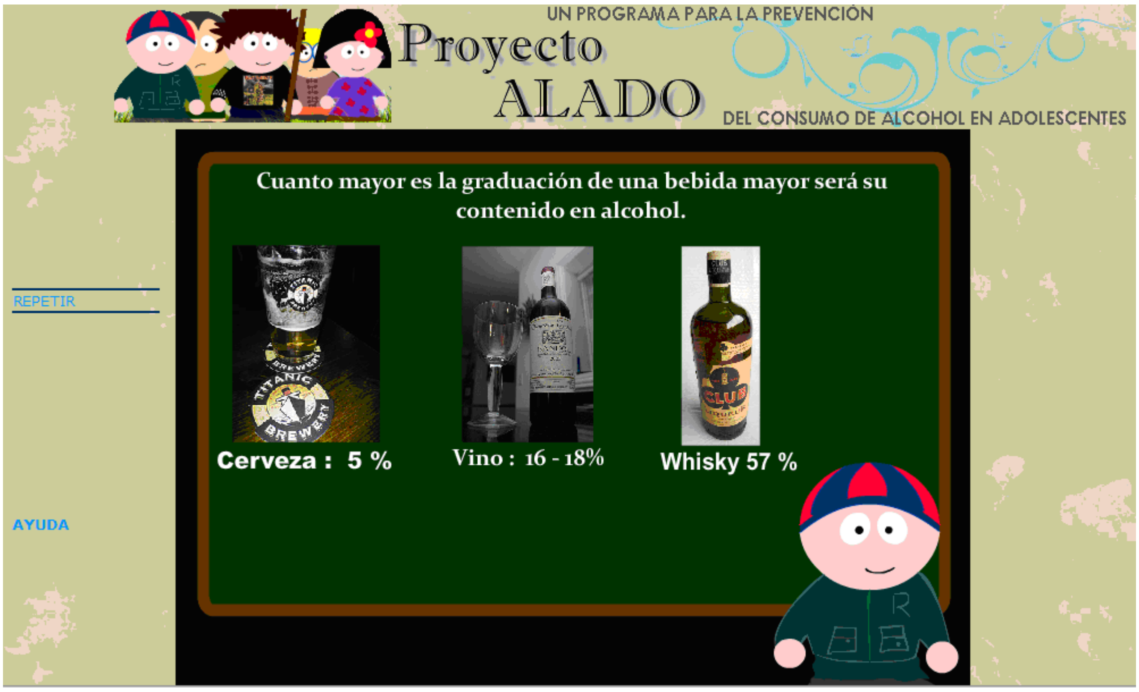

Figure 1A. Examples of sequences of concepts and principles about the effects of alcohol on immature brains

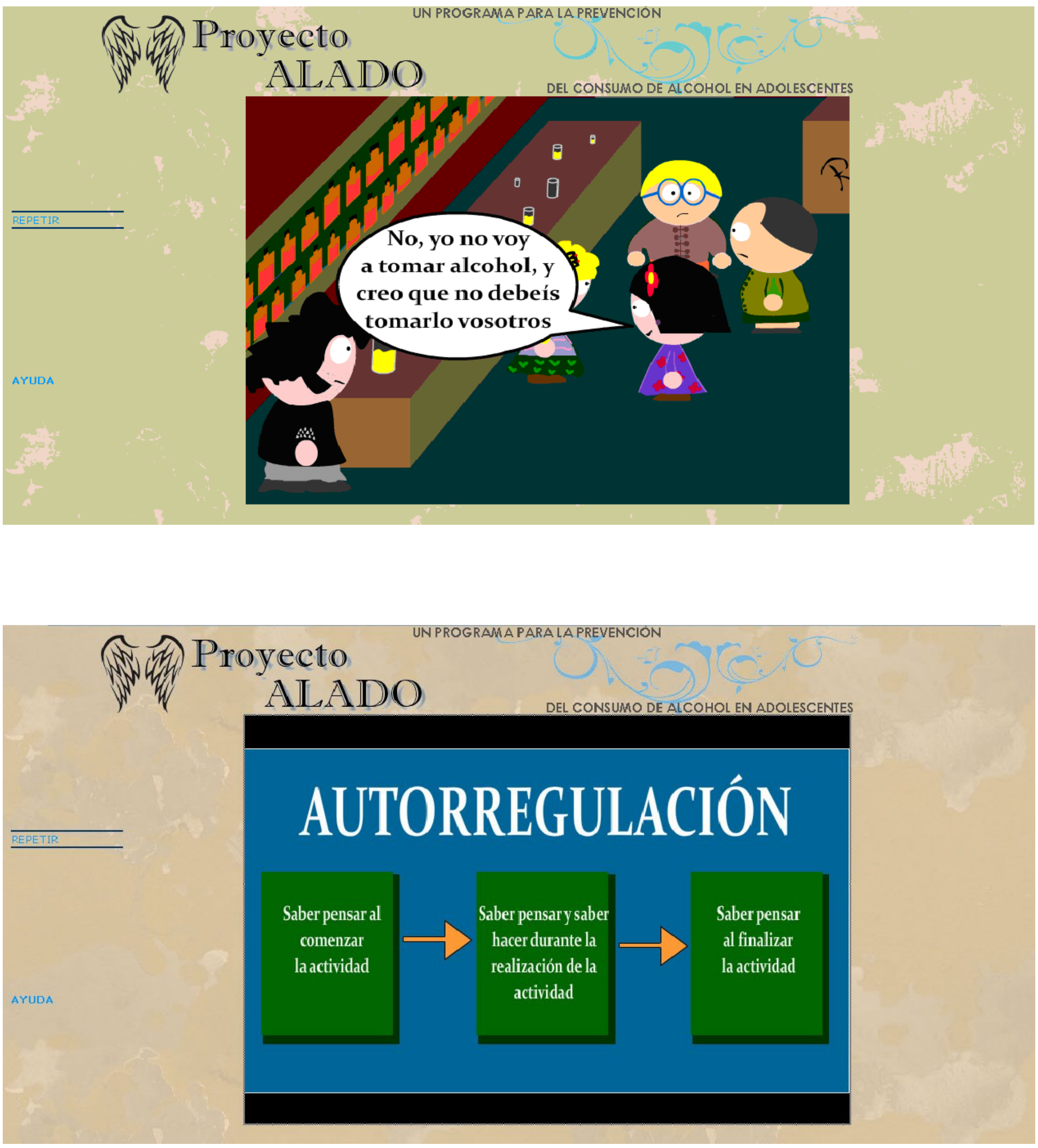

Figure 2A. Examples of sequence of assertive skills and self-regulation in alcohol consumption

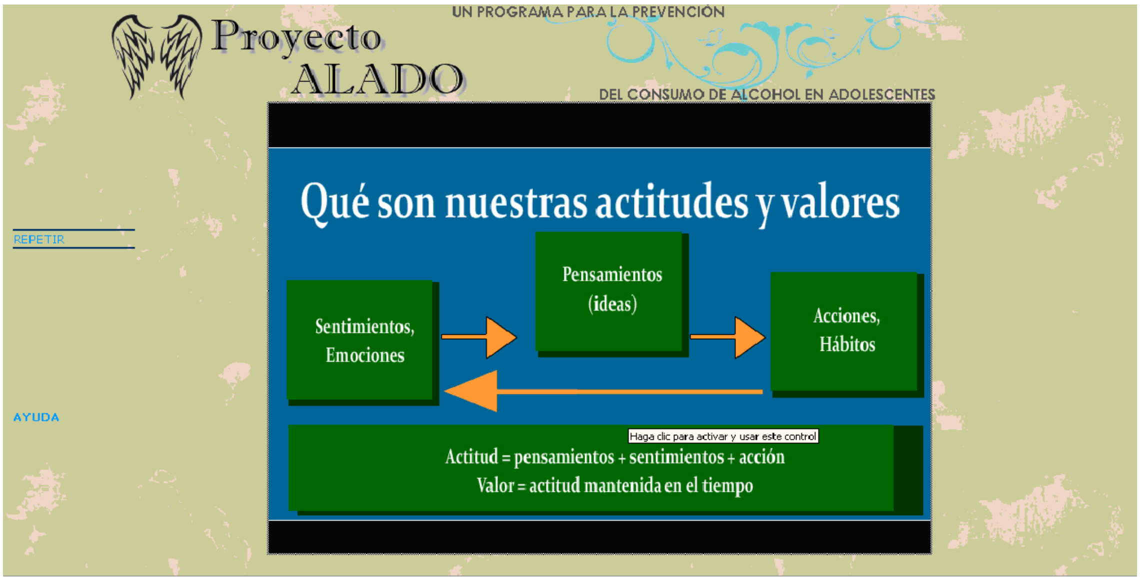
UN PROGRAMA PARA LA PREVENCIÓN
Proyecto ALADO
DEL CONSUMO DE ALCOHOL EN ADOLESCENTES
REPETIR
AYUDA
Qué son nuestras actitudes y valores
Pensamientos (ideas)
Sentimientos, Emociones
Acciones, Hábitos
Haga clic para activar y usar este control
Actitud = pensamientos + sentimientos + acción
Valor = actitud mantenida en el tiempo

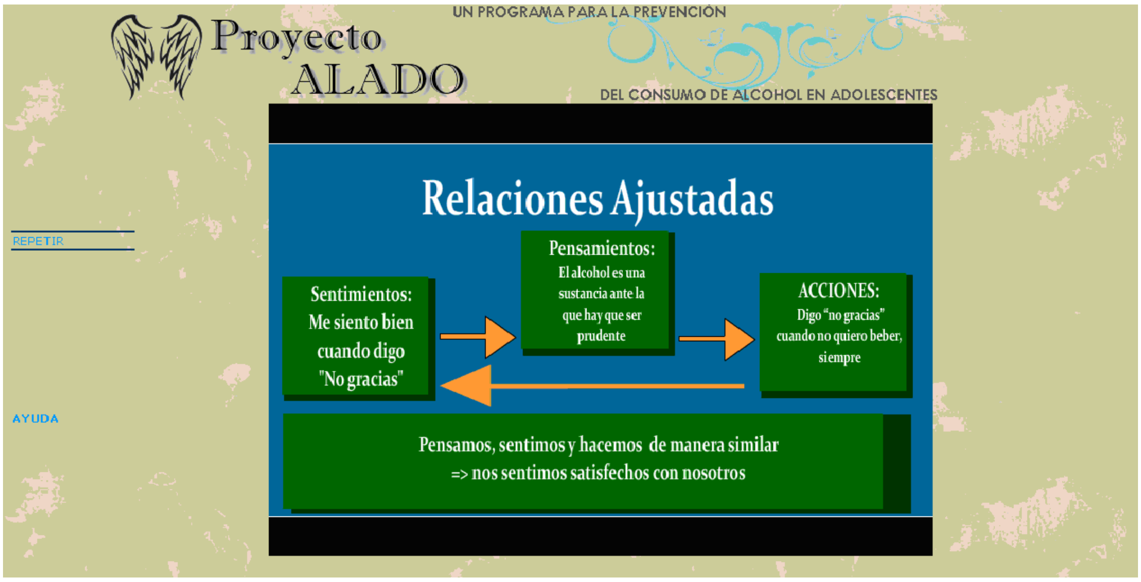
UN PROGRAMA PARA LA PREVENCIÓN
Proyecto ALADO
DEL CONSUMO DE ALCOHOL EN ADOLESCENTES
REPETIR
AYUDA
Relaciones Ajustadas
Pensamientos: El alcohol es una sustancia ante la que hay que ser prudente
Sentimientos: Me siento bien cuando digo "No gracias"
ACCIONES: Digo "no gracias" cuando no quiero beber, siempre
Pensamos, sentimos y hacemos de manera similar
=> nos sentimos satisfechos con nosotros

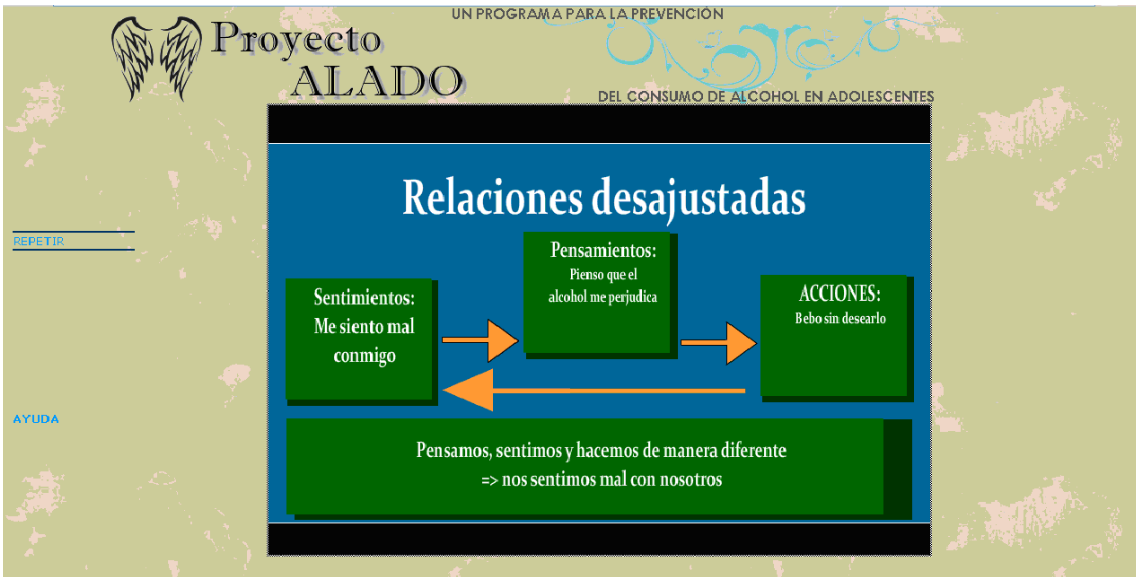

Figure 3A. Examples of sequence of behavioral components of attitudes applied to alcohol consumption
